# Acrylate Functionalized Tetraalkylammonium Salts with Ionic Liquid Properties

**DOI:** 10.3390/molecules17066593

**Published:** 2012-05-31

**Authors:** Dorian C. Grothe, Wolfdietrich Meyer, Silvia Janietz

**Affiliations:** Fraunhofer Institute for Applied Polymer Research, Geiselberg Str. 69, 14476 Potsdam, Germany

**Keywords:** ionic liquid, room temperature molten salt, electrolytes

## Abstract

Acrylate functionalized ionic liquids based on tetraalkylammonium salts with terminal acrylates- and methylacrylates were synthesized. Melting points and ionic conductivity of twenty compounds in six groups were determined. Within one group the effect of three different counterions was investigated and discussed. The groups differ in cationic structure elements because of their functional groups such as acrylate and methacrylate, alkyl residues at the nitrogen and number of quaternary ammonium atoms within the organic cation. The effect of these cationic structure elements has been examined concerning the compiled parameters with a view to qualifying them as components for solid state electrolytes. The newly synthesized ionic liquids were characterized by NMR and FTIR analysis. The exchange of halide ions like bromide as counter ions to weakly coordinating [PF_6_]^−^, [OTf]^−^ or [TFSI]^−^ reduces the melting points significantly and leads to an ion conductivity of about 10^−4^ S/cm at room temperature. In the case of the dicationic ionic liquid, an ion conductivity of about 10^−3^ S/cm was observed.

## 1. Introduction

The development of new energy storage devices like batteries demands electrolytes that fulfill today’s performance and security requirements. These two qualities would seem to be incompatible with each other in today’s battery separators that allow ionic transport from cathode to anode, as either high ionic conductivity or high thermal and electrochemical stability is available. Fluid electrolytes guarantee ionic conductivities of 0.01 S/cm at room temperature, but consist of organic solvents with boiling points above 240 °C, true for the most common solvents such as propane carbonate (PC) and ethylene carbonate (EC). Still, these solvents also are volatile and exhibit a risk of fire and deflagration [1,2].

Therefore the development of new non-volatile and non-flammable ion conducting materials is a challenge to enhance the safety and lifetime of the energy storage devices. Ionic liquids are interesting candidates for this application. Ionic liquids are defined as “organic molten salts” with a melting point below 100 °C but more often they are desired as room temperature ionic liquids which are in a liquid state at ambient temperature [3,4]. Due to their unique properties like thermal- and chemical stability and negligible vapor pressure, they have received much attention and were studied extensively in the 80s with a focus on using them as solvents or electrolytes for batteries [5,6,7].

Today most of the known and investigated room temperature ionic liquids are of the monocation type with the imidazole, pyridinium or tetralkylammonium cation structures [8,9,10]. As counter ions, usually triflates [OTF]^−^, hexafluorophosphate [PF_6_]^−^ tetrafluoroborate [BF_4_]^−^ or recently bis(trifluoromethanesulfonyl)imide [TFSI]^−^ are used [11].

The groups of Ohno and Cardiano recently showed a new type of ionic liquid, which contains a polymerizable group, e.g., an acrylate group, and an alkyl spacer to an imidazolium ionic liquid to analyze the effect of terminal cationic structure for the ionic conductivity for further use in energy devices like lithium batteries [12,13]. Another special sort of ionic liquid is the dication type which was described recently by the groups of Zhang and Lall [14,15]. The dicationic ionic liquids have a higher charge density and thus may be favorable for applications as electrolytes.

In our work we focus on a special sort of ionic liquids; ionic liquids with the capability to polymerize. We wanted to investigate the influence of the structure for ionic conductivity and melting behavior and test if this class of material is appropriate for further use in blend systems for lithium ion batteries. Therefore we synthesized ionic liquids based on the tetraalkyllammonium type with different alkyl chain lengths and different counter ions. In addition we combined both aspects: The one of the previous mentioned polymerizable and the dicationic ionic liquids and created a new polymerizable dicationic ionic liquid, which in contrast to the already reported dicationic ionic liquids, has an asymmetric shape [16]. We expected that the higher charge density of the dication would lead to higher ion conductivity and a lower melting point. Also the asymmetric shape of the ionic liquid may lead to an amorphous polymer structure which would be favorable for future applications like solid state electrolytes.

## 2. Results and Discussion

**Scheme 1:**
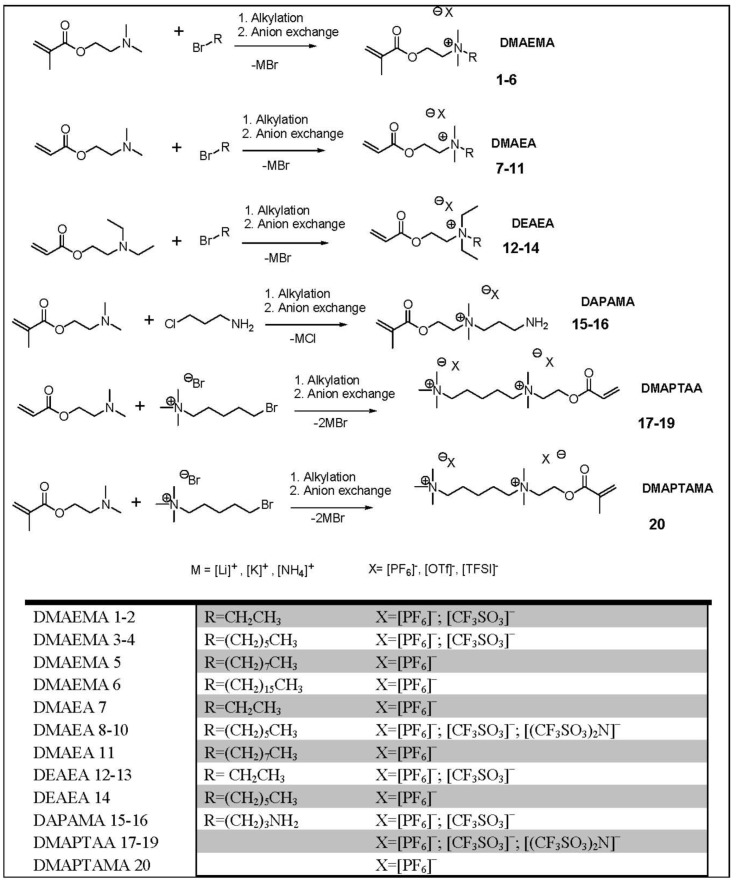
General two-step synthesis leading to polymerizable ionic liquids.

In this work we have investigated the influence of the cation structure, especially the alkyl chain length at the nitrogen; the anion structure and the influence of the polymerizable group for the physical properties. We investigated in detail melting points and conductivities in relation to the structure of the unpolymerized ionic liquid. The following synthetic route was used for the polymerizable ionic liquids shown in Scheme 1. 

The quaternization reaction of the amine with an alkyl halide produces the ionic liquid. In a second step an anion exchange of the halide ions through salt metathesis with [PF_6_]^−^, [CF_3_SO_3_]^−^ and [(CF_3_SO_3_)_2_N]^−^ was done. The alkylation at the nitrogen of DMAEMA and DMAEA occured with higher yields (around 90%) when it was done under inert atmosphere and with heating (50 °C). 

### 2.1. Melting Points

**Figure 1:**
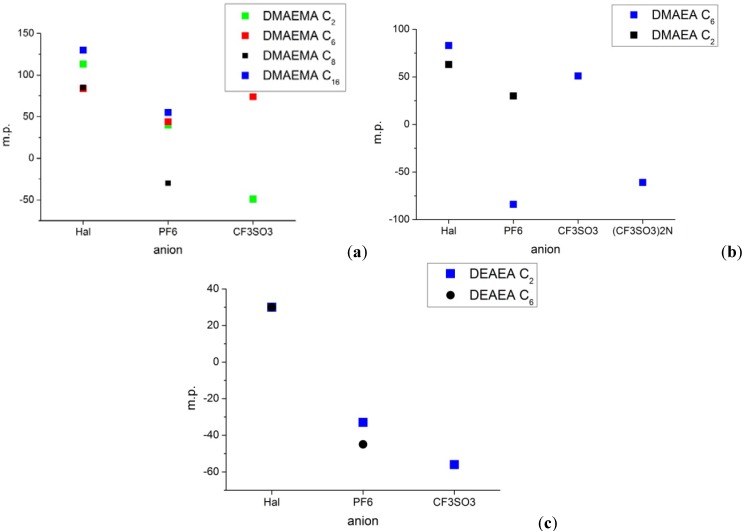
Melting point *vs.* anion (**a**) DMAEMA with C_2_ to C_16_ alkyl chain length; (**b**) DMAEA with C_2 _and C_6_ alkyl chain length; (**c**) DEAEA with C_2_ and C_6_ alkyl chain length.

Table 1 lists the measured melting points. It is seen that methacrylates have higher melting points than acrylates (compare Figure 1a). The additional methyl group arranges the monomers in a way which favors crystallization. The exchange of methyl groups for ethyl groups at the nitrogen reduces the melting points as well, due to steric hindrance. It also lowers the formation of the quaternary ammonium salts significantly. 

**Table 1:** Melting points and conductivity.

Ionic liquid monomer	Melting Point in °C	Conductivity in liquid state in S/cm at RT
DMAEMA 1	40	5.86E-06
DMAEMA 2	−49	4.86E-04
DMAEMA 3	44	2.50E-04 *
DMAEMA 4	74	1.25E-05 **
DMAEMA 5	−30	1.71E-04
DMAEMA 6	57	1.37E-05 *
DMAEA 7	70	3.03E-04 **
DMAEA 8	−84	4.20E-04
DMAEA 9	51	2.63E-05 *
DMAEA 10	−61	7.14E-04
DMAEA 11	<−100	4.95E-05
DEAEA 12	−33	1.41E-04
DEAEA 13	−56	3.80E-04
DEAEA 14	−45	5.65E-06
DAPAMA 15	−36	3.33E-05
DAPAMA 16	−34	1.95E-04
DMAPTAA 17	<−100	2.20E-03
DMAPTAA 18	−43	1.90E-04
DMAPTAA 19	−49	4.24E-04
DMAPTAMA 20	<−100	7.30E-04

* Measured at 60 °C; ** Measured at 100 °C.

The choice of anion has a much greater impact on the melting point. A weakly coordinating anion like hexafluorophosphate reduces the melting point dramatically. Due to its great steric demand bis(trifluoromethane sulfonyl)imide almost always leads to an amorphous structure.

The introduction of small alkyl chains like ethyl groups prevents crystallization at moderate temperatures. In contrast, the attachment of long alkyl chains like hexadecyl groups enables the possibility to crystallize along the alkyl chain. It turns out that an alkyl chain length in the range of four to eight carbon atoms is able to form ionic liquids at room temperature.

The dicationic ionic liquids have a relatively high melting point with halides as counter ions. After the anion exchange the melting point drops significantly. For the case of hexafluorophosphate we could not observe any crystallization via DSC measurement down to −100 °C.

### 2.2. Ion Conductivity

**Figure 2:**
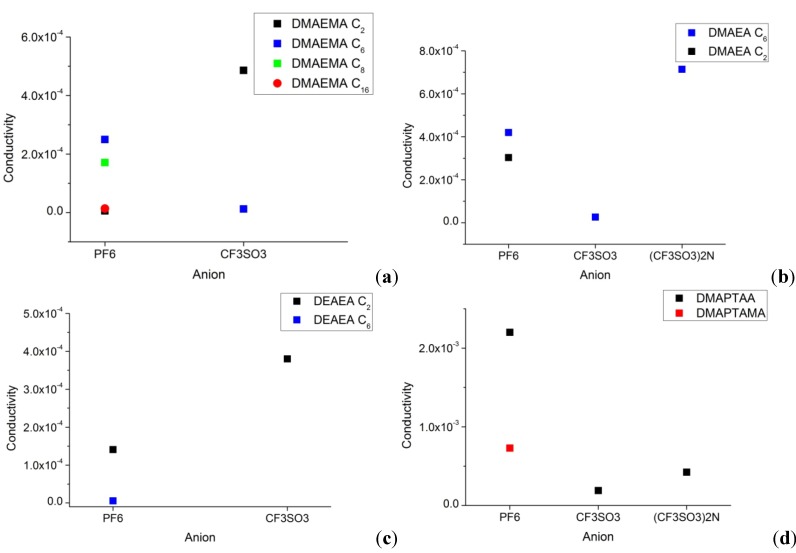
Conductivity *vs.* anion (**a**) DMAEMA with C_2_ to C_16_ alkyl chain length. (**b**) DMAEA with C_2 _and C_6_ alkyl chain length. (**c**) DEAEA with C_2_ and C_6_ alkyl chain length. (**d**) Dicationic ionic liquids.

The ion conductivity is an essential property for further use of ionic liquids, for example as a substitute for common electrolyte solvents. The conductivity depends on the viscosity. A low viscosity almost always results in high ion conductivity. Therefore the bis(trifluoromethanesulfonyl)imide anion is favorable [7]. As expected the dicationic type ionic liquids has a higher ionic conductivity due to higher charge density in the molecule (Table 1 and Figure 2).

## 3. Experimental

### 3.1. General

#### 3.1.1. Materials

1-Bromoethane, 1-bromohexane, 1-bromohexadecane, potassium triflate, bis (trifluoromethane)-sulfonamide lithium salt and (5-bromopentyl)-trimethylammonium bromide were purchased from Aldrich. Dimethylaminoethyl acrylate and dimethylaminoethyl methacrylate were received from Alfa Aesar. Diethylaminoethyl methylacrylate and potassium hexaflurophosphate were purchased from ABCR-Chemicals. These chemicals were used as received without further purification.

#### 3.1.2. Solvents

Chloroform was freshly distilled over calcium chloride. Acetone, dry acetonitrile stored over molecular sieves, and diethyl ether were used as received without further drying. All solvents were purchased from Roth. 

#### 3.1.3. Measurements

The structure of the introduced ILs was confirmed by ^1^H-NMR spectroscopy (INOVA 500 spectrometer, Varian, Germany) and FTIR-Spectroscopy (Scimitar 2000 FT-IR from Varian, Germany). Melting points were measured with a Stuart-SMP10 melting point apparatus. The conductivity of RTIL was confirmed with a Mettler Toledo Seven multi-modular meter (Germany).

#### 3.1.4. Synthesis

The synthesis procedure of the polymerizable ionic liquids was, according to the works by Altomare *et al.* and Ohno *et al.*, divided into two steps: first the formation of the dication via quaternization reaction at the nitrogen and then the conversion into the desired ionic liquid through anion exchange was done [3,17,18]. 

### 3.2. Synthesis of Monocation Type Ionic Liquids

[2-(Methacryloyloxy) ethyl] * dimethylethylammonium bromide* (**DMAEMA 1–2**). A mixture of 2,2-dimethylaminoethyl methylacrylate (10.07 g, 64.05 mmol) and 1-bromoethane (7.03 g, 64.51 mmol) were dissolved in distilled chloroform and heated under inert atmosphere at 55 °C for 24 h. After removing all volatile components the yellowish residue was washed with diethyl ether. The resulting precipitate was collected and washed again with diethyl ether. The obtained white solid then was dried under vacuum yielding 16.04 g (94.1%) of the product. M.p.: 113 °C; ^1^H-NMR (CDCl_3_) δ (ppm): 1.40 (t, 3H, CH_3_); 1.95 (s, 3H, CH_3_); 3.20 (s, 6H, CH_3_–N); 3.60 (m, 2H, CH_2_–N); 3.85 (m, 2H, CH_2_–N); 4.60 (m, 2H, CH_2_–O); 5.65 (s, 1H, vinyl-CH); 6.10 (s, 1H, vinyl-CH; IR (cm^−1^): 955 cm^−1^δ(–(CH_2_)_n_;1019 δ(CH_2_–CH_3_); 1154 ν_s_(C–O–C); 1319 ν_as_(C–O–C); 1457 δ_s_(CH_3_); 1633 ν(C=C); 1712 ν(C=O); 3003 ν(C–H).

[2-(Methacryloyloxy) hexyl] * dimethylethylammonium bromide* (**DMAEMA 3–4**). A mixture of 2,2-dimethylaminoethyl methylacrylate (10.02 g, 63.74 mmol) and 1-bromohexane (10.51 g, 63.67 mmol) were dissolved in dest. chloroform and vigorously stirred under inert atmosphere at 50 °C for 24 h. The removal of all volatile components results in a waxy white solid which then was recrystallized from acetone and washed with diethyl ether. The obtained white solid then was dried under vacuum yielding 16.94 g (82.6%) of the product. M.p.: 84 °C; ^1^H-NMR (CDCl_3_) δ (ppm): 0.95 (t, 3H, CH_3_); 1.35 (m, 6H, CH_2_); 1.75 (m, 2H, CH_2_); 1.95 (s, 3H, CH_3_); 3.55 (s, 6H, CH_3_–N); 3.65 (m, 2H, CH_2_–N); 4.15 (m, 2H, CH_2_–N); 4.70 (m, 2H, CH_2_–O); 5.70 (s, 1H, vinyl-CH); 6.15 (s, 1H, vinyl-CH; IR (cm^−1^): 747 cm^−1^δ(–(CH_2_)_n_, n > 4); 1153 ν_s_(C–O–C); 1307 ν_as_(C–O–C); 1461 δ_s_(CH_3_); 1636 ν(C=C); 1719 ν(C=O); 2938 ν(C–H).

[2-(Methylacyloyloxy)octyl]*diemthylethylammonium bromide* (**DMAEMA 5**). A mixture of 2,2-dimethylaminoethyl methylacrylate (2.80 g, 17.79 mmol) and 1-bromooctane (3.66 g, 18.90 mmol) were dissolved in dest. chloroform and vigorously stirred under inert atmosphere at 50 °C for 24 h. After the removal of all volatile components under reduced pressure, the residue was washed and precipitated with diethyl ether. The resulting solid was collected via filtration and dried under vacuum yielding 3.88 g (62.3%) of a white solid. M.p.: 85 °C; ^1^H-NMR (CDCl_3_) δ (ppm): 0.95 (t, 3H, CH_3_); 1.20–1.40 (m, 10H, CH_2_); 1.75 (m, 2H, CH_2_); 1.95 (s, 3H, CH_3_); 3.55(s, 6H, CH_3_–N); 3.65 (m, 2H, CH_2_–N); 4.15 (m, 2H, CH_2_–N); 4.65 (m, 2H, CH_2_–O); 5.70 (s, 1H, vinyl-CH); 6.15 (s, 1H, vinyl-CH); IR (cm^−1^): 723 cm^−1^δ(–(CH_2_)_n_, n > 4); 1154 ν_s_(C–O–C); 1318 ν_as_(C–O–C); 1391 δ_s_(CH_3_); 1463 δ_as_(CH_3_); 1635 ν(C=C); 1720 ν(C=O); 2922 ν(C-H).

[2-(Methylacyloyloxy)hexadecyl]*diemthylethylammonium bromide* (**DMAEMA 6**). A mixture of 2,2-dimethylaminoethyl methylacrylate (3.72 g, 23.66 mmol) and 1-bromohexadecane (7.30 g, 23.90 mmol) were dissolved in dist. chloroform and vigorously stirred under inert atmosphere at 50 °C for 24 h. After the removal of all volatile components under reduced pressure, the residue was washed and precipitated with diethyl ether. The resulting solid was collected via filtration and dried under vacuum yielding 9.03 g (82.5%) of a white solid. M.p.: 130 °C; ^1^H-NMR (CDCl_3_) δ (ppm): 0.95 (t, 3H, CH_3_); 1.20–1.40 (m, 26H, CH_2_); 1.75 (m, 2H, CH_2_); 1.95 (s, 3H, CH_3_); 3.20(s, 6H, CH_3_–N); 3.35 (m, 2H, CH_2_–N); 3.75 (m, 2H, CH_2_–N); 4.55 (m, 2H, CH_2_–O); 5.65 (s, 1H, vinyl-CH); 6.15 (s, 1H, vinyl-CH; IR (cm^−1^): 723 δ(–(CH_2_)_n_, n > 4); 1166 ν_s_(C–O–C); 1300 ν_as_(C–O–C); 1463 δ_s_(CH_3_); 1636 ν(C=C); 1719 ν(C=O); 2916 ν(C–H).

[2-(Acryloyloxy)ethyl]*dimethylethylammonium bromide* (**DMAEA 7**). A mixture of 2,2-dimethyl-aminoethyl acrylate (1.40 g, 9.84 mmol) and 1-bromoethane (1.07 g, 9.85 mmol) were dissolved in dest. chloroform and vigorously stirred under inert atmosphere at 50 °C for 24 h. After the removal of all volatile components under reduced pressure the remaining residue was washed several times with diethyl ether. The resulting yellowish gel was recrystallized from acetone and washed again with diethyl ether to get an orange-white solid. After drying under vacuum 1.66 g (67.2%) of the desired product was obtained. M.p.: 73 °C; ^1^H-NMR (CDCl_3_) δ (ppm): 1.45 (t, 3H, CH_3_); 3.50 (s, 6H, N–CH_3_); 3.85 (m, 2H, N–CH_2_); 4.15 (m, 2H, N–CH_2_); 4.70 (m, 2H, O–CH_2_); 5.95 (d, 1H, vinyl-CH); 6.15 (m, 1H; vinyl-CH), 6.45(m, 1H, vinyl-CH). IR (cm^−1^): 965 δ(–(CH_2_)_n_; 1049 δ (CH_2_–CH_3_); 1176 ν_s_(C–O–C); 1262 ν_as_(C–O–C); 1456 δ_as_(CH_3_); 1632 ν(C=C); 1725 ν(C=O); 2974 ν(C–H).

[2-(Acryloyloxy)hexyl]dimethylethylammonium* bromide* (**DMAEA 8–10**). A mixture of 2,2-dimethyl-aminoethyl acrylate (14.08 g, 105.50 mmol) and 1-bromohexane (17.70 g, 107.22 mmol) were dissolved in dist. chloroform and vigorously stirred under inert atmosphere at 50 °C for 24 h. After the removal of all volatile components under reduced pressure, the residue was washed and precipitated with diethyl ether. The resulting solid was collected via filtration and dried under vacuum yielding 31.07 g (95.9%) of a yellow-white solid. M.p.: 83 °C; ^1^H-NMR (CDCl_3_) δ (ppm): 0.95 (t, 3H, CH_3_); 1.3(m, 6H, CH_2_); 1.75 (m, 2H, CH_2_); 3.50 (s, 6H, CH_3_–N); 3.65 (m, 2H, CH_2_–N); 4.15 (m, 2H, CH_2_–N); 4.70 (m, 2H, CH_2_–O); 5.95 (d, 1H, vinyl-CH); 6.15 (dd, 1H, C=CH–CO); 6.50 (d, 1H, vinyl-CH); IR (cm^−1^): 734 cm^−1^δ(–(CH_2_)_n_, n > 4); 1178 ν_s_(C–O–C); 1293 ν_as_(C–O–C); 1410 δ_s_(CH_3_); 1466 δ_as_(CH_3_); 1636 ν(C=C); 1727 ν(C=O); 2959 ν(C-H).

[2-(Acryloyloxy)octyl]*dimethylethylammonium bromide* (**DMAEA 11**). A mixture of 2,2-dimethyl-aminoethyl acrylate (2.80 g, 19.69 mmol) and 1-bromooctane (4.00 g, 20.69 mmol) were dissolved in dist. chloroform and vigorously stirred at 50 °C under inert atmosphere for 24 h. After removal of all volatile components under reduced pressure, the residue was washed several times with diethyl ether. The obtained orange oil was then dried under vacuum yielding 4.54 g (69.0%) of the desired product. ^1^H-NMR (CDCl_3_) δ (ppm): 0.95 (t, 3H, CH_3_); 1.25–1.35 (m, 10H, CH_2_); 1.75 (m, 2H, CH_2_); 3.15 (s, 6H, CH_3_–N); 3.35 (m, 2H, CH_2_–N); 3.75 (m, 2H, CH_2_-N); 4.55 (m, 2H, CH_2_-O); 5.95 (d, 1H, vinyl-CH); 6.15 (dd, 1H, C=CH-CO); 6.45 (d, 1H, vinyl-CH); IR (cm^−1^): 749 cm^−1^δ(–(CH_2_)_n_, n > 4); 1186 ν_s_(C–O–C); 1280 ν_as_(C–O-–C); 1444 δ_s_(CH_3_); 1467 δ_as_(CH_3_); 1644 ν(C=C); 1728 ν(C=O); 2927 ν(C–H).

[2-(Acryloyloxy)ethyl]*diethylethylammonium bromide* (**DEAEA 12–13**). A mixture of 2,2-diethyl-aminoethyl acrylate (6.45 g, 37.67 mmol) and 1-bromoethane (4.38 g, 40.19 mmol) were dissolved in dry acetone and vigorously stirred under inert atmosphere at 50 °C for 24 h. After the removal of all volatile components under reduced pressure, the residue was washed several times with diethyl ether. The obtained orange oil then was dried under vacuum yielding 3.68 g (34.9%) of the desired product.^1^H-NMR (CDCl_3_) δ (ppm):1.35 (m, 9H, CH_3_); 3.45 (m, 6H, CH_2_–N); 3.70 (m, 2H, CH_2_–N); 4.55 (m, 2H, CH_2_–O); 5.95 (d, 1H, vinyl-CH); 6.15 (dd, 1H, C=CH–CO); 6.45 (d, 1H, vinyl-CH). 

[2-(Acryloyloxy)hexyl]*diethylethylammonium bromide* (**DEAEA 14**). A mixture of 2,2-diethyl-aminoethyl acrylate (10.00 g, 58.40 mmol) and 1-bromohexane (11.00 g, 66.63 mmol) were dissolved in dist. chloroform and vigorously stirred under inert atmosphere at 50 °C for 24 h. After the removal of all volatile components under reduced pressure, the residue was washed and precipitated several times with diethyl ether. The resulting solid was collected via filtration and dried under vacuum yielding 0.40g (2.1%) of a beige-white solid. M.p.: 83 °C; ^1^H-NMR (CDCl_3_) δ (ppm): 0.95 (t, 3H, CH_3_); 1.30–1.40 (m, 14H, CH_2_, N–CH_2_–CH_3_); 1.70 (m, 2H, CH_2_); 3.25 (m, 4H, CH_2_–N); 3.45 (m, 2H, CH_2_–N); 3.70 (m, 2H, CH_2_–N); 4.60 (m, 2H, CH_2_–O); 5.95 (d, 1H, vinyl-CH); 6.15 (dd, 1H, C=CH–CO); 6.45 (d, 1H, vinyl-CH); IR (cm^−1^): 804 δ(–(CH_2_)_n_, n > 4); 1070 δ (CH_2_–CH_3_); 1188 ν_s_(C–O–C); 1266 ν_as_(C–O–C); 1409 δ_s_(CH_3_); 1456 δ_as_(CH_3_); 1624 ν(C=C); 1722 ν(C=O); 2959 ν(C–H).

[2-(Methacryloyloxy)propylamine]*dimethylethylammonium chloride* (**DAPAMA 15–16**). A mixture of chloropropylamine hydrochloride (4.13 g, 31.79 mmol) and dimethylaminoethyl methylacrylate (4.85 g, 30.82 mmol) were dissolved in dry acetonitrile and vigorously stirred under inert atmosphere at 60 °C for 48 h. After the removal of the solvent under reduced pressure the residue was dissolved in chloroform, the precipitated solid was removed via filtration. After removing all remaining volatile components the residue was washed several times with diethyl ether and then dried under vacuum yielding 5.76 g (74.5%) of a yellow-brown gel. M.p.: 20 °C; ^1^H-NMR (CDCl_3_) δ (ppm): 1.35 (m, 2H, CH_2_); 1.95 (s, 3H, CH_3_); 2.95 (s, 6H, N–CH_3_); 3.20 (m, **2**H, N–CH_2_); 3.40 (m, 2H, N–CH_2_); 3.75 (m, 2H, N–CH_2_); 4.40 (m, 2H, O–CH_2_); 5.65 (m, 1H, vinyl-CH); 6.10 (m, 1H; vinyl-CH).

### 3.3. Synthesis of Dication Type Ionic Liquids

*5-[(2-Acryloyloxyethyl)-dimethylammonium]-pentyltrimethylammonium bromide* (**DMAPTAA**). A mixture of (5-bromopentyl)-trimethylammonium bromide (1.44 g, 4.99 mmol) and dimethyl-aminoethyl acrylate (0.74 mL, 4.86 mmol) were dissolved in dry acetonitrile and vigorously stirred under inert atmosphere at 50 °C for 48 h. After the removal of all volatile components under reduced pressure the residue was washed and precipitated several times with diethyl ether. The resulting yellow white solid was collected via filtration and dried under vacuum yielding 1.6 g of the desired product (76.3%). M.p.: 100 °C; ^1^H-NMR (CD_3_OD) δ (ppm): 1.55 (t, 2H, CH_2_); 1.95 (m, 4H, CH_2_); 3.15 (m, 4H, CH_2_–N); 3.25 (s, 15H, CH_3_), 3.55 (m, 2H, CH_2_–N);4.65 (m, 2H, CH_2_–O); 6.05 (d, 1H, vinyl-CH); 6.25 (dd, 1H, vinyl-CH); 6.50 (d, 1H, vinyl-CH); IR (cm^−1^): 809 δ(–(CH_2_)_n_, n > 4); 1025δ (CH_2_–CH_3_); 1185 ν_s_(C–O–C); 1267 ν_as_(C–O–C); 1407 δ_s_(CH_3_); 1480 δ_as_(CH_3_); 1629 ν(C=C); 1723 ν(C=O); 2952 ν(C-H).

*5-[(2-Methylacryloyloxyethyl)dimethylammonium]-pentyltrimethylammonium bromide* (**DMAPTAMA**). A mixture of (5-bromopentyl)trimethylammonium bromide (1.19 g, 4.12 mmol) and dimethylaminoethyl methacrylate (0.69 mL, 4.09 mmol) were dissolved in acetonitrile and vigorously stirred under an inert atmosphere at 60 °C for 48 h. After the removal of all volatile components under reduced pressure the residue was washed and precipitated several times with diethyl ether. The resulting white solid was collected via filtration and dried under vacuum yielding 1.75 g of the desired product (95.9%). M.p.: 158 °C; ^1^H-NMR (CD_3_OD) δ (ppm): 1.55 (t, 2H, CH_2_); 1.90 (m,4H, CH_2_); 1.95 (s, 3H, CH_3_); 3.15 (m, 4H, CH_2_–N); 3.25 (s, 15H, CH_3_), 3.55 (m, 2H, CH_2_–N);4.65 (m, 2H, CH_2_–O); 6.05 (d, 1H, vinyl-CH); 6.25 (dd, 1H, vinyl-CH); 6.50 (d, 1H, vinyl-CH); IR (cm^−1^): 847 δ(–(CH_2_)_n_, n > 4); 1031δ (CH_2_–CH_3_); 1161 ν_s_(C–O–C); 1296 ν_as_(C–O–C); 1480 δ_as_(CH_3_); 1634 ν(C=C); 1718 ν(C=O); 2953 ν(C–H).

### 3.4. Anion Exchange

#### 3.4.1. Monocation Type

The anion exchange was done by the common method of salt metathesis. 

**[PF_6_]^−^**: The ammonium halide salt was dissolved in chloroform and a solution of potassium hexafluorophosphate in acetone was added at 45 °C. This solution was stirred for 24 h and the precipitated potassium halide was removed via filtration. The reaction solution then was washed with deionised water several times until the water phase was free from any halide traces as checked with silver nitrate. After the removal of the solvent the respective product was dried under vacuum for 24 h.

**[CF_3_SO_3_]^−^/[(CF_3_SO_3_)_2_N]^−^**: The ammonium halide salt was dissolved in chloroform adding a solution of potassium triflate/lithium bis(trifluoromethanesulphonyl)imide in methanol. This solution was stirred at 45 °C for 24 h. The precipitated halide salt was removed via filtration. After removal of the solvent the residue was washed with deionized water until the washed solution was free from halide traces.

#### 3.4.2. Dication Type

The ammonium halide salt was dissolved in methanol adding a solution of potassium triflate/ lithium bis(trifluoromethanesulphonyl)imide in methanol respectively in acetone for potassium hexafluorophosphate. This solution was stirred at 45 °C for 24 h. The precipitated halide salt was removed via filtration. After the removal of the solvent the residue was dissolved in acetonitrile, insoluble components were removed via filtration. The solvent was removed under reduced pressure and the residue was washed with deionized water until the washed solution showed no trace of halide ions through silver nitrate test. The obtained product was then dried under vacuum for 24 h.

## 4. Conclusions

In this work we have demonstrated a simple route for the synthesis of polymerizable ionic liquids via quaternization- and anion exchange reactions. We also could show that the cation structure has an influence on the melting point of ionic liquids, but the choice of anion is of much greater importance. It was demonstrated also that the conductivity of the ionic liquids corresponds to their melting point/viscosity. With this information it should be easier to design future ionic liquids for special use such as materials for solid state electrolytes. From these investigated structure-property correlations the compounds **DMAEA 8–10** and **DMAPTAA 17–19** from the synthesized polymerizable ionic liquids are chosen as components for solid-state-electrolytes. Their application as components in solid-state-electrolytes will be presented in a future paper.
